# Microbial-derived salt-tolerant proteases and their applications in high-salt traditional soybean fermented foods: a review

**DOI:** 10.1186/s40643-023-00704-w

**Published:** 2023-11-18

**Authors:** Hongli Yao, Shuangping Liu, Tiantian Liu, Dongliang Ren, Zhilei Zhou, Qilin Yang, Jian Mao

**Affiliations:** 1 National Engineering Research Center of Cereal Fermentation and Food Biomanufacturing, School of Food Science and Technology, Jiangnan University, Wuxi, 214122 Jiangsu China; 2 Southern Marine Science and Engineering Guangdong Laboratory, Guangzhou, 511458 Guangdong China; 3 Jiangsu Provincial Engineering Research Center for Bioactive Product Processing Technology, Jiangnan University, Wuxi, 214122 Jiangsu China; 4 Jiangnan University (Shaoxing) Industrial Technology Research Institute, Shaoxing, 31200 Zhejiang China; 5 National Engineering Research Center of Huangjiu, Zhejiang Guyuelongshan Shaoxing Wine CO., LTD, Shaoxing, 646000 Zhejiang China; 6 Department of Biology and Food Engineering, Bozhou University, Bozhou, 236800 Anhui China

**Keywords:** Application, Mechanism, Microorganism, Modern biotechnology, Salt tolerance, Salt-tolerant protease, Soy fermentable foods

## Abstract

Different microorganisms can produce different proteases, which can adapt to different industrial requirements such as pH, temperature, and pressure. Salt-tolerant proteases (STPs) from microorganisms exhibit higher salt tolerance, wider adaptability, and more efficient catalytic ability under extreme conditions compared to conventional proteases. These unique enzymes hold great promise for applications in various industries including food, medicine, environmental protection, agriculture, detergents, dyes, and others. Scientific studies on microbial-derived STPs have been widely reported, but there has been little systematic review of microbial-derived STPs and their application in high-salt conventional soybean fermentable foods. This review presents the STP-producing microbial species and their selection methods, and summarizes and analyzes the salt tolerance mechanisms of the microorganisms. It also outlines various techniques for the isolation and purification of STPs from microorganisms and discusses the salt tolerance mechanisms of STPs. Furthermore, this review demonstrates the contribution of modern biotechnology in the screening of novel microbial-derived STPs and their improvement in salt tolerance. It highlights the potential applications and commercial value of salt-tolerant microorganisms and STPs in high-salt traditional soy fermented foods. The review ends with concluding remarks on the challenges and future directions for microbial-derived STPs. This review provides valuable insights into the separation, purification, performance enhancement, and application of microbial-derived STPs in traditional fermented foods.

**Graphical Abstract:**

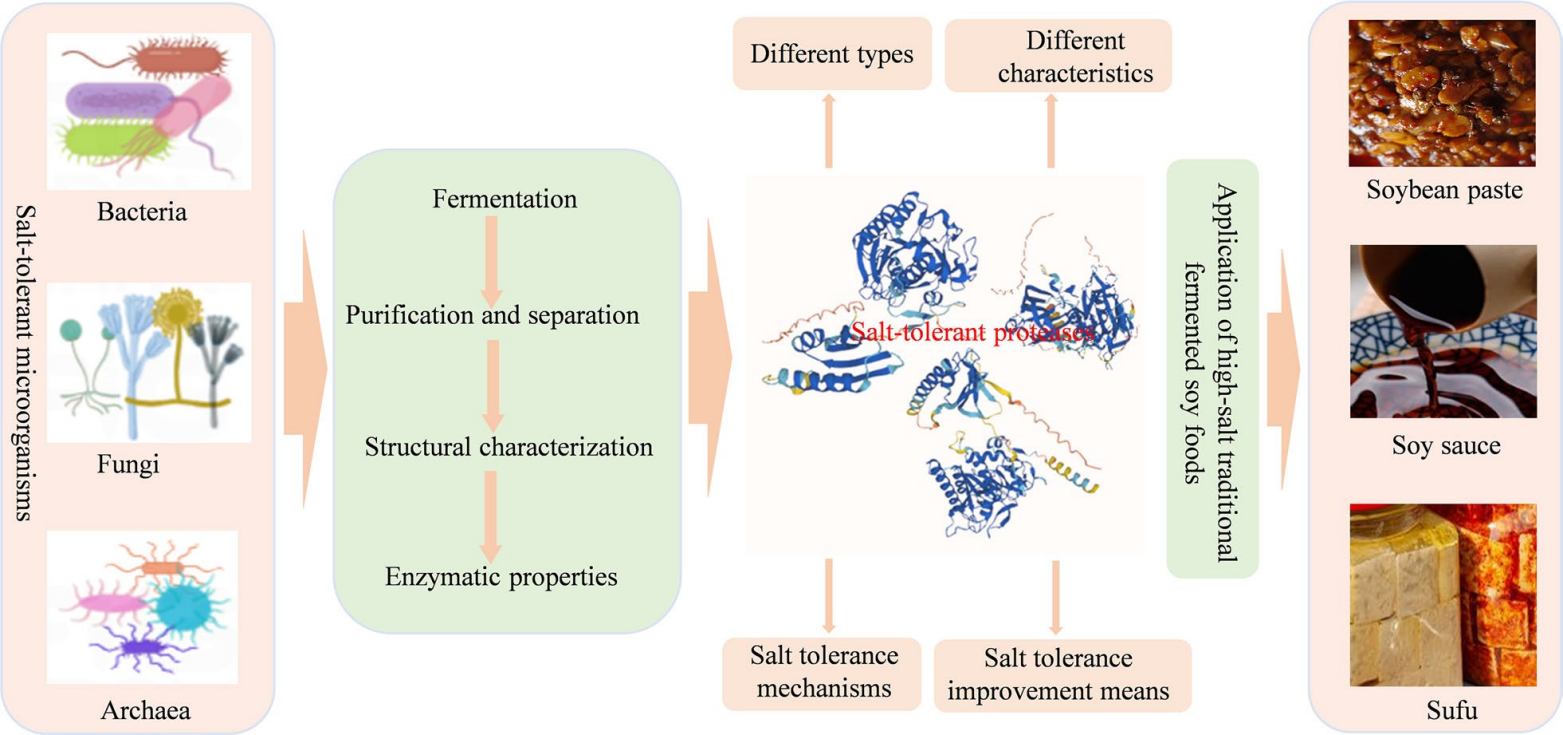

## Introduction

Proteases are a class of enzymes that hydrolyze proteins, breaking them down into smaller peptides and amino acids (Aguilar and Sato 2018; Razzaq et al. 2019). Depending on the peptide bond hydrolyzed, proteases can be divided into exopeptidases, which cleave the N- or C-terminal peptide bond, and endopeptidases, which cleave the internal peptide bond (Boon et al. 2020; Matkawala et al. 2021). Depending on the optimal pH for their action, they can be classified as alkaline, acidic, or neutral proteases (Matkawala et al. 2021). Additionally, they can be divided into serine proteases, sulfhydryl proteases, metalloproteases, and acid proteases, according to the group in which they exert their hydrolytic action (Matkawala et al. 2021). Remarkably, certain proteases demonstrate high selectivity by selectively hydrolyzing specific peptide bonds in protein substrates. Microorganisms, including bacteria, fungi, and archaea, have the ability to secrete various types of proteases (Aguilar and Sato 2018; Razzaq et al. 2019). For example, the bacterium *Bacillus cereus* is known to secrete alkaline proteases (Orhan et al. 2005), while the fungus *Aspergillus niger* is capable of secreting acidic proteases (Wei et al. 2023). These microbial-derived proteases possess advantageous characteristics, including high efficiency, stability, and ease of purification. Consequently, they are widely employed in various industries such as food, feed, pharmaceutical, and leather processing (Chanalia et al. 2011; Matkawala et al. 2021; Rao et al. 1998).

Due to their own variability, microorganisms may be able to produce proteases that are adapted to different pH values, temperatures, pressures, and other industrial requirements (Naveed et al. 2021). In some specific environments (such as high salt), common proteases tend to lose their activity, which limits their application. Salt-tolerant proteases (STPs) are known for their high salt tolerance, and are able to withstand concentrations of approximately 3.0 M NaCl or higher (Gao et al. 2019, 2018). They are often found in organisms adapted to high-salt environments. STPs are more promising for industrial applications due to their higher salt tolerance, wider adaptability, and more efficient catalytic ability under extreme conditions compared to common proteases. In the food processing industry, STPs can be used in high-salt foods, such as soy sauce, curd, shrimp paste, and sausages (Gao et al. 2019, 2018; Naveed et al. 2021). Their contribution lies in their ability to hydrolyze proteins, thereby improving the taste, flavor, and overall quality of such foods. In the environmental industry, STPs can help improve water quality by breaking down complex organic compounds in industrial wastewater into simpler, more easily removable forms (Anh et al. 2021; Sivaprakasam et al. 2011). In addition, STPs have shown potential as antifungal agents in agriculture, food production, and biotechnology (Essghaier et al. 2009). They have also been used in detergent formulations for the effective removal of blood, egg, grass, and ketchup stains (Mokashe et al. 2017). Given their distinctive functional properties and potential commercial value, STPs have attracted considerable attention from researchers.

In recent years, there has been an increasing number of studies on microbial-derived STPs. These studies have focused on screening or identifying novel salt-tolerant microorganisms from different ecological environments using various modern techniques to uncover novel STPs with superior properties (Gao et al. 2020; Nishikawa et al. 2022; Sun et al. 2020; Takenaka et al. 2022). The catalytic mechanisms of STPs have been intensively studied using molecular biology and protein chemistry techniques to reveal their unique catalytic properties in high-salt environments (Gao et al. 2019). Using protein modification and computer-aided simulation techniques, researchers have successfully modified protease molecules to enhance the salt tolerance of the enzyme itself (Senba et al. 2023; Takenaka et al. 2022; Takita et al. 2008). Novel STP preparations have been developed and are being tested for applications in biopharmaceuticals, food processing, environmental treatment, and other fields (Fu et al. 2008; Hao et al. 2022; Zhang et al. 2019). In addition, optimizing the growth conditions of enzyme-producing microorganisms and developing effective isolation and purification protocols are important research directions for researchers to obtain novel STPs with potential functional value and capable of meeting industrial demands (Setyorini et al. 2006; Sivaprakasam et al. 2011; Vidyasagar et al. 2007). However, despite numerous scientific reports on the discovery of novel STPs, improvement in STP activity and yield, exploration of STP mechanisms, and expansion of commercial applications (An et al. 2023; Banerjee and Ray 2017; Karan et al. 2012; Mokashe et al. 2018), a systematic review of microbial-derived STPs and their application to traditional high-salt soy-based fermented foods is notably absent. This comprehensive review introduces the STP-producing microbial species and their selection methods, and summarizes and analyzes the salt tolerance mechanisms of the microorganisms. It also presents different methods for the isolation and purification of STPs and discusses the mechanisms underlying their salt tolerance. In addition, it highlights the significant contribution of modern biotechnology in enhancing salt tolerance in STPs. Importantly, this review emphasizes the wide range of applications of STPs in high-salt traditional soybean fermented foods and offers insights into the future development of microbial-derived STPs. By consolidating this information, this review aims to provide a valuable resource for researchers to identify gaps in knowledge concerning the process of isolating and purifying microbial-derived STPs, improving their performance, and exploring their applications in traditional fermented foods.

## Study on microorganisms producing STPs

### Microorganisms producing STPs

Microorganisms producing STPs primarily include bacteria, fungi, and some archaea. Table 1 collects information on some microbial-derived STPs reported since 2015. Notably, the strains have been isolated from various high-salt environments such as saline soils, seawater, lakes, and marine sediments, and used in various industries (Daoud et al. 2018; Flores-Santos et al. 2020; Foophow et al. 2022; Yang et al. 2020). STPs produced by different microorganisms differ not only in salt tolerance, but also in structure and enzymatic properties such as the optimum temperature, pH, and metal ion inhibition. These differences in structure and properties directly influence the range of industrial applications for these STPs (Chung et al. 2022; Falkenberg et al. 2022; Yang et al. 2020). The alkaline protease from the halotolerant alkaliphilic *Bacillus* sp. strain NPST-AK_15_ can remain stable in NaCl up to 20% (w/v). The optimum pH and temperature for the enzyme are 10.5 and 60 °C, respectively. When pre-incubated with some commercial detergents at 40 °C for 1 h, it retains the initial activity of 75–100%. These results suggest that it may play a significant role in commercial detergents (Ibrahim et al. 2015a). Similarly, alkaline proteases used in detergent formulations have been reported to be active at concentrations up to 4 M NaCl, with enhanced activity in the presence of CaCl_2_, KCl, and MnCl_2_ (Mokashe et al. 2017). However, the optimum pH and temperature are 8.5 and 55 °C, respectively, which are lower than those of the alkaline protease from *Bacillus* sp. strain NPST-AK_15_. Most of the reported STPs from microorganisms are alkaline proteases, while reports of salt-tolerant neutral proteases and acid proteases are few (Table 1). The salt-tolerant neutral protease from *Aspergillus oryzae* CICIM F0899 has shown high salt tolerance in 18% NaCl and can maintain 72% of its initial activity after 14 days (Wang et al. 2013). It has promising applications in soy sauce production. The acid protease from *Rhodotorula mucilaginosa* L7 can maintain 60% activity in 3.5 M NaCl concentration, and its optimum pH and temperature are 5.0 and 50 °C, respectively (Lario et al. 2015). This protease may lead to potential biotechnological applications.

**Table 1:** Some STPs from microorganisms and their potential applications reported in recent 8 years

Microbial source	Microbial type	Microbial name	Type of protease	Potential application	References
Hypersaline soda lakes	Bacterium	*Bacillus* sp. NPST-AK_15_	Alkaline serine protease	Laundry and pharmaceutical industries	Ibrahim et al. 2015a
Seawater of South China Sea	Bacterium	*Pseudoalteromonas* sp. 129-1	Alkaline protease	Laundry detergent and non-toxic anti-biofilm agent	Wu et al. 2015
Hypersaline lake	Bacterium	*Bacillus iranensis* strain X5B	Halo-alkaline serine protease	–	Ghafoori et al. 2016
Antarctic marine alga	Yeast	*Rhodotorula mucilaginosa* L7	Acid protease	Biotechnology industry	Lario et al. 2015
Water and sediment samples	Bacterium	*Bacillus agaradhaerens* AK-R	Alkaline protease	Laundry and pharmaceuticals industries	Ibrahim et al. 2016
Mixed water–sediment samples	Bacterium	*Alkalibacillus* sp. NM-Da2	Alkalithermophilic protease	Biotechnology and pharmaceutical industries	Abdel-Hamed et al. 2016
Fish sauce	Fungus	*Penicillium citrinum* YL-1	Alkaline serine protease	Fish sauce fermentation	Xie et al. 2016
Çankırı salt mine and Lake Tuz	Archaeon	*Haloarcula* sp. TG_1_	Acid protease	Biotechnology industry	Abanoz et al. 2017
Chinese marine solar salterns	Archaeon	*Halogranum rubrum*	Alkaline protease	Fish sauce	Gao et al. 2017
Saline soil	Bacterium	*Salinicoccus* sp. UN-12	Alkaline Protease	Detergent	Mokashe et al. 2017
Garden soil	Fungus	*Aspergillus niger* WA 2017	Alkaline protease	The silver recovery from used X-ray film	Wahab and Ahmed 2018
Guangdong Institute of Microbiology	Fungus	*Aspergillus oryzae* 3.042	Alkaline protease	Fermented soybean food	Gao et al. 2019
Guangdong Institute of Microbiology	Fungus	*A. oryzae* 3.042	Aspartyl aminopeptidase	Fermented soybean products	Gao et al. 2018
The top-layer of the highly saline soda lime	Bacterium	*Bacillus luteus* H11	Halo-alkaline protease	Food, detergent, environmental bioremediation, and pharmaceutical industries	Kalwasinska et al. 2018
Marine sediment samples	Bacterium	*Vibrio* sp. LA-05	Metalloprotease	Detergent formulations	Zhang et al. 2018
Saline and high-salt soil and water	Bacterium	*Bacillus halodurans* strain US_193_	Halo-alkaline protease	Detergents	Daoud et al. 2018
Salted fish	Bacterium	*Virgibacillus* sp. CD6	Protease	Detergents	Lam et al. 2018
Egyptian soda lakes	Bacterium	*Salipaludibacillus agaradhaerens* strain AK-R	Alkaline serine protease	Laundry detergent industry	Ibrahim et al. 2019
Seawater	Bacterium	*Barrientosiimonas* sp. V_9_	Extremophilic proteases	Laundry, leather processing, medicine, food, and waste management	Flores-Santos et al. 2020
Fish sauce	Bacterium	*Bacillus velezensis* SW_5_	Alkaline serine protease	Medical treatment	Yang et al. 2020
Shrimp paste and Fresh shrimp	Bacterium	*Virgibacillus halodenitrificans* ST-1	Protease	Fermentation of shrimp paste	Liu et al. 2020
Sediments of the Lake Oubeïra	Bacterium	*Gracilibacillus boraciitolerans* strain LO15	Serine alkaline protease	Detergent	Ouelhadj et al. 2020
The rocks	Haloarcheon	*Halococcus* sp. strain GUGFAWS-3	Metalloproteinase	Various biotechnological and bioremediation applications	Gaonkar and Furtado 2020
The Okha site	Actinomycete	*Nocardiopsis alba* strain OM- 5	Alkaline serine protease	Detergent, bioremediation, and food	Chauhan et al. 2021
China General Microbiological Culture Collection Center	Archaeon	*Halococcus salifodinae*	Serine protease	Laundry detergents, leather products, pharmaceuticals, diagnostics and food products	Hou et al. 2021
Mangrove forest sediments	Bacterium	*Bacillus licheniformis* KB111	Halo-alkaline proteases	–	Foophow et al. 2022
German collection of microorganisms and cell cultures GmbH	Bacterium	*Alkalihalobacillus okhensis* Kh10-101^ T^	Alkaline subtilisin	Biotechnology industry	Falkenberg et al. 2022
Sea water from Coastal Gujarat and Diu in the Western India	Bacterium	*Haloalkaliphilic bacterium* D-15–9	Alkaline proteases	Detergent, leather processing, silver recovery, pharmaceutical usage, food processing, peptide synthesis, and waste treatment	Raval et al. 2022
Sea water from Coastal Gujarat and Diu in the Western India	Bacterium	*Oceanobacillus onchorynchii* Mi-10-5_4_	Alkaline proteases	Detergent, leather processing, silver recovery, pharmaceutical usage, food processing, peptide synthesis, and waste treatment	Raval et al. 2022
Seawater	Fungus	*Aspergillus reticulatus* strain SK1-1	Halophilic proteases	Food processing, detergents, textiles, and waste treatment	Chung et al. 2022

– represents that no relevant information is involved in the reference

Table 1

Halophytic bacteria, known for their ability to thrive and reproduce in high-salt environments, have been extensively studied for their ability to produce STPs. These bacteria include a wide variety of genera, such as *Bacillus*, *Halophilus*, *Pseudomonas*, *Vibrio*, *Halococcus*, *Salinobacterium*, *Nocardia*, and *Salipaludibacillus* (Lee et al. 2010). Among these, *Bacillus* stands out as an important source of STPs, and a large number of purified and characterized STPs originate from this genus (Ibrahim et al. 2015b). A new halotolerant alkaliphilic strain of *Bacillus*, specifically *Bacillus* sp. strain NPST-AK_15_, has been isolated from a high-salt lake (Ibrahim et al. 2015b). This strain has shown potential to produce an extracellular serine protease, which further expands the range of STP species available for study and application. A recently discovered extracellular haloprotease has been successfully isolated and purified from *Bacillus licheniformis* KB111 (Foophow et al. 2022). With their distinctive enzymatic properties, these proteases have found a wide range of applications in diverse industries including detergents, leather production, food processing, medicine, textiles, and environmental protection. For instance, the alkaline serine protease isolated and purified from *Bacillus velezensis* SW_5_ exhibits remarkable fibrinolytic activity, which is expected to achieve efficient fibrin hydrolysis in medical treatment (Yang et al. 2020). Additionally, a novel halogen- and heat-resistant alkaline protease derived from *Bacillus agaradhaerens* strain AK-R has been purified, which may have potential applicability in laundry and pharmaceutical operations due to its exceptional tolerance to temperature, pH, and salt concentration (Ibrahim et al. 2016).

Fungi represent a significant source of STPs, with notable species including *A. niger*, *A. oryzae*, *Aspergillus reticularis*, *Penicillium citrus*, and *Penicillium nalgiovense* (Foophow et al. 2022; Lee et al. 2010). Many of these fungus-derived STPs have demonstrated potential applications in traditional high-salt fermented foods, such as soy sauce, shrimp paste, and fish paste. One promising example is the halotolerant alkaline serine protease derived from *Penicillium citrinum* YL-1, which exhibits superior salt tolerance compared to proteases from other bacteria known for their high salt tolerance. This characteristic makes it a promising candidate for the hydrolysis of fish protein during fish sauce fermentation (Xie et al. 2016). *A. oryzae* 3.042, a strain isolated from traditional high-salt foods, has been found to produce not only alkaline proteases (Gao et al. 2019) but also salt-tolerant neutral protease (Wang et al. 2013), acid protease (Lee et al. 2010), and aspartate aminopeptidase (Gao et al. 2018). These enzymes play a crucial role in the utilization of fermented food proteins and the production of flavor compounds. Similarly, the fungus *A. niger*, capable of producing these enzymes simultaneously, has been employed in high-salt traditional foods alongside *A. oryzae* to enhance product quality and flavor (Leng and Xu 2011; Wahab and Ahmed 2018; Wang et al. 2021c).

Archaea has emerged as a novel and promising source of STPs. With increasing research on archaea, it has been discovered that many of these microorganisms can produce STPs. These archaea include *Haloarcula*, *Halococcus*, and others (Abanoz et al. 2017; Gaonkar and Furtado 2020; Hou et al. 2021). *Haloarcula* sp. TG_1_, isolated from salt mines and salt lakes, has been found to produce proteases with maximum activity at pH 4.0, 50 °C, and 4 M NaCl (Abanoz et al. 2017). Moreover, *Halococcus agarilyticus* GUGFAWS-3, isolated from a marine white sponge, can produce two halo-extremozymes: protease and lipase. The optimal activity of the protease is pH 7.0, 70 °C, and 3 M NaCl. Notably, this represents the first report of halophilic neutral protease production within the *Halococcus* genus (Gaonkar and Furtado 2020). Although the production of STPs in archaea has been less extensively studied than in other microorganisms, the investigation of extracellular proteases in salt-tolerant archaea holds great potential to expand the field of protease applications and contribute to the exploration of novel STPs.

### Screening methods for microorganisms producing STPs

Traditional methods for selecting STP-producing microorganisms rely primarily on the specific microbial production environment. These environments include highly saline habitats such as oceans, salt lakes, salt farms, saline soils, and salt-containing foods. Isolation of salt-tolerant microorganisms from these environments requires many steps such as sample collection, isolation of strains, morphological characteristics and molecular sequence identification of strains (Abanoz et al. 2017). To obtain excellent STP-producing strains, additional steps such as optimizing enzyme production conditions and evaluating the enzyme production capacity of the microorganism based on the enzyme activity index have been implemented (Wahab and Ahmed 2018; Zhang et al. 2023). While microorganisms obtained through conventional screening methods exhibit high salt tolerance and stable enzyme production capacity, limitations such as low screening efficiency and time consumption may restrict their widespread application.

In order to obtain organisms with enhanced protease production capacity, various methods have been employed to screen for strains exhibiting high levels of STP production, including UV mutagenesis, chemical reagent mutagenesis, room-temperature plasma mutagenesis, and genetic engineering. For example, by mutagenesis of *A. oryzae* 3.042 at room temperature and atmospheric pressure, a mutant strain, designated H8, has been obtained with significant activity of neutral protease, alkaline protease, and aspartyl aminopeptidase (Gao et al. 2020). The incorporation of this strain in soy sauce fermentation processes has led to significant improvements in peptide and amino acid content. The serine protease gene *isp* from *Bacillus* sp. LCB_10_ has been cloned and expressed in *Escherichia coli* using genetic engineering techniques. The resulting serine protease demonstrates high tolerance to NaCl, exhibiting stable activity in the presence of 1–5% NaCl and retaining 86% of its activity in the presence of 7% NaCl (Hou et al. 2019). Additionally, the gene encoding the extracellular protease, *sptA*, has been cloned from the halophilic archaeon *Natrinema* sp. J7 and successfully expressed in *Haloferax volcanii* WFD11 (Shi et al. 2006). The resultant recombinant enzyme exhibits remarkable stability, retaining activity even in the presence of 2.5 M NaCl at 50 °C and pH 8.0. While these methods significantly enhance the likelihood of attaining excellent protease-producing strains, certain challenges such as screening limitations and demanding technical requirements are also encountered. Screening processes relying on wild-type strains may suffer from screening blind spots and involve high workloads.

**Fig. 1:**
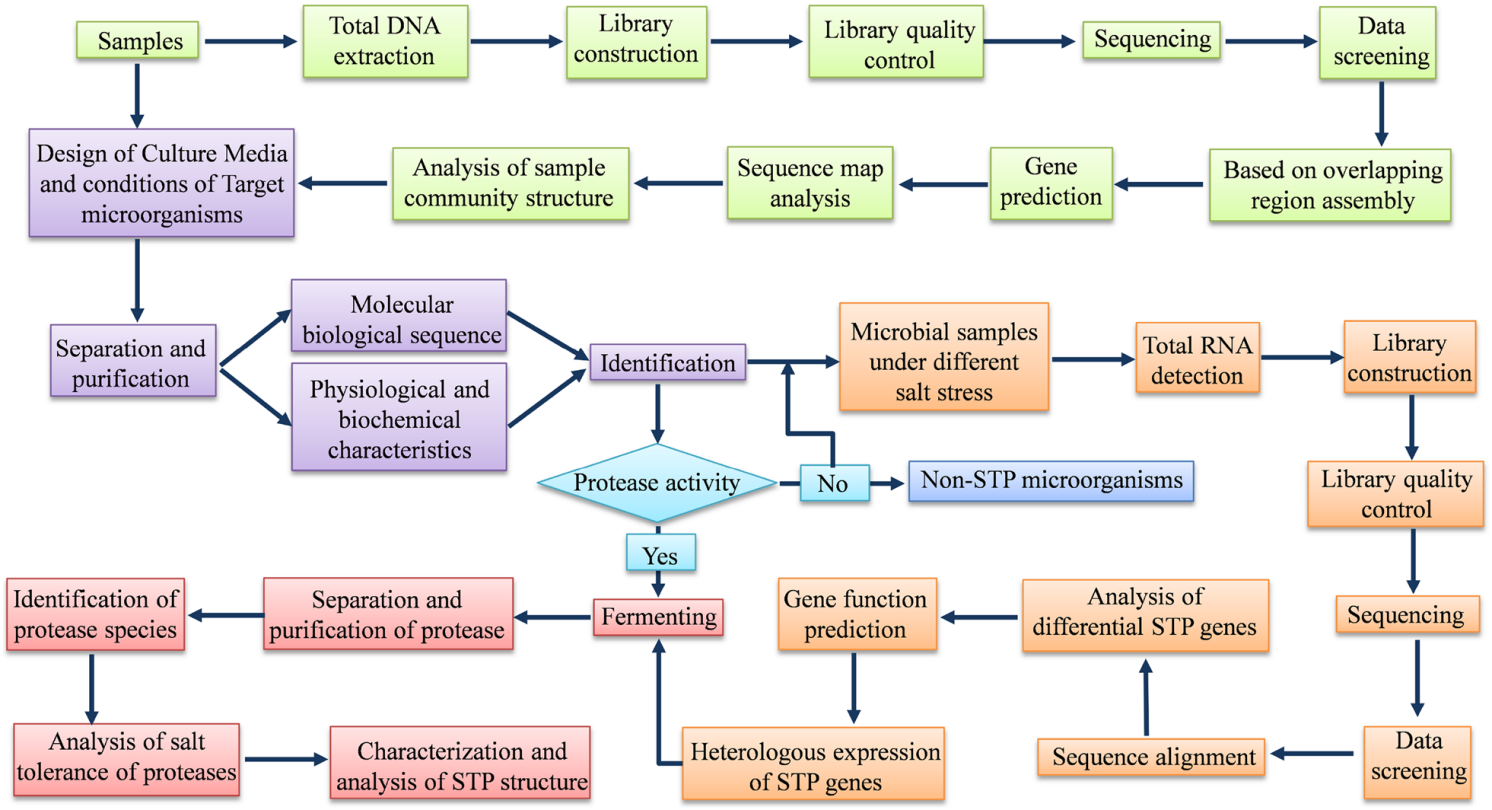
A process framework for screening STP-producing microorganisms using multi-omics techniques

In nature, microorganisms are categorized as either culturable and unculturable, especially those living in extreme environments, which may produce STPs with special structures and functions (Wang et al. 2021a). To increase the likelihood of discovering novel secondary metabolites even from unculturable microorganisms, metagenomics is employed to analyze the functional potential of DNA extracted from environmental samples. For instance, a metagenomic library has been constructed from the DNA of the Chumathang hot spring sediment, leading to the identification of heat-resistant, basophilic, and oxidative-stable serine proteases (Singh et al. 2015). Using a metagenomic screening approach, another serine protease gene has been cloned from a saline habitat and is active in saline conditions (Purohit and Singh 2013). A novel thermostable serine protease has been obtained from a metagenomic library derived from marine sediments in the East China Sea, which not only shows good thermal activity and stability, but also has strong salt tolerance (40% residual activity when kept in 3 M NaCl) (Sun et al. 2020). With the advancement of bioinformatics, there has been a growing number of studies on integrated metagenomic approaches such as metagenomics and metaproteomics to assist in the screening of STP-producing microorganisms (Busche et al. 2018; Garcia-Moyano et al. 2021; Yang et al. 2022). Using metagenomics and peptidomics analysis, 138 umami peptides and 6 kinds of proteases from 35 microbial genera have been identified from traditional fish fermentation (Yang et al. 2022). The role of cellular proteases in *Streptomyces* protein secretion has been identified by multi-omics and targeted approaches, providing the first multi-omics techniques effort to characterize the complex regulatory mechanisms of *Streptomyces* protein secretion (Busche et al. 2018). Multi-omics techniques can help scientists to fully understand the genetic, transcriptional, protein, and metabolic information of microorganisms, thereby aiding microbial breeding and optimization (Aguiar-Pulido et al. 2016; Ge et al. 2021; Wu et al. 2022). A process framework for screening STP-producing microorganisms using multi-omics techniques is shown in Fig. 1. Based on gene, protein and enzyme activity, microorganisms capable of producing STPs can be rapidly screened from a large number of samples using modern bioinformatics techniques. In summary, the breeding of STP-producing microorganisms requires the combined use of physical, chemical, biological, and information technologies, as well as other technological means. In the future, it is worth discussing how these techniques can be used to quickly, accurately, and efficiently select microorganisms producing STPs.

### Salt-tolerant mechanisms of microorganisms

**Fig. 2:**
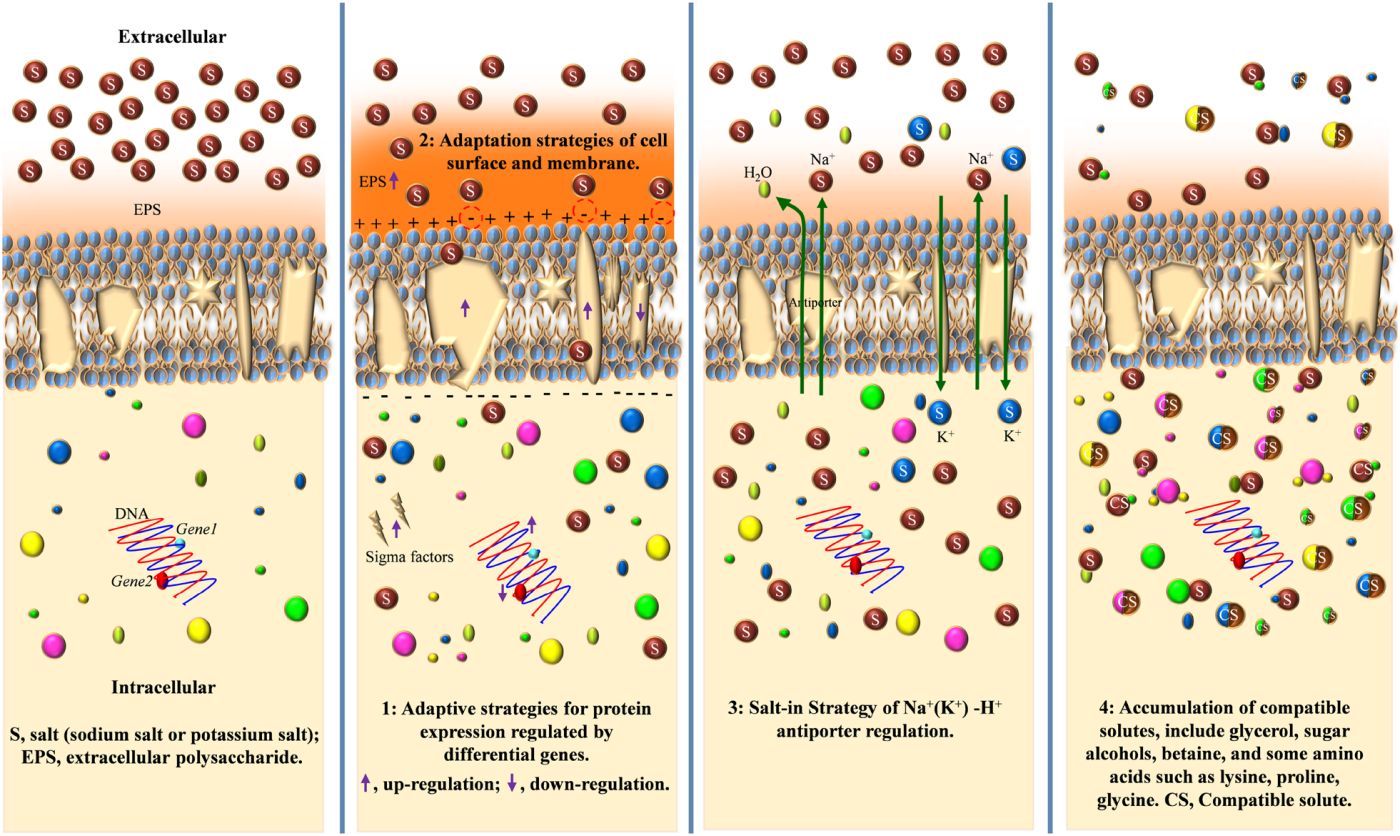
Several salt tolerance mechanisms of some salt-tolerant bacteria

Microorganisms can adapt to a wide range of NaCl concentrations, and their NaCl tolerance may be far greater than that of any other organism (Vreeland 1987). Halophilic/halotolerant microorganisms that are able to hydrolyze proteins are an important source of STPs. Halophilic/halotolerant microorganisms are well adapted to higher salt concentrations, and the enzymes they produce must remain active in high-salinity environments (Mokashe et al. 2018). Several studies have been reported on the salt-tolerant mechanisms of microorganisms (Chen et al. 2021; Kurt-Kizildogan et al. 2017). Halophilic microorganisms prefer high-salt concentrations to survive and reproduce compared to halotolerant microorganisms, but this review does not strictly distinguish the salt-tolerant mechanisms between halophilic and halotolerant microorganisms. In general, these microorganisms adapt to different salt concentrations through rejection, secretion, and intracellular accumulation, and these processes have been attributed to multiple mechanisms (Edbeib et al. 2016; Vreeland 1987). Several salt tolerance mechanisms for some salt-tolerant bacteria are shown in Fig. 2. These salt-tolerant mechanisms are summarized below.

Adaptive strategies employed by microorganisms for achieving salt tolerance involve the differential expression of gene regulatory proteins. Under the stress of a highly osmotic environment, these microorganisms regulate and orchestrate protein expression by up-regulating, down-regulating, and replicating pertinent genes. Such proteins include membrane transporters, oxidative stress proteins, ferritin, as well as various enzymes, such as dehydrogenase, synthase, and salt-tolerant enzymes (Chen et al. 2021; Soppa 2006). A transcriptional analysis of low- and high-salt genes in the extremely halophilic archaea *Halolalama* sp. YKT1, isolated from a salt mine in Turkey, has revealed that 2149 genes are up-regulated and 1638 genes are down-regulated under high-salt conditions (Kurt-Kizildogan et al. 2017). Notably, the upregulation of genes associated with membrane transporters, the CRISPR–Cas system, osmotic protection solutes, oxidative stress proteins, and iron metabolism is closely linked to salt tolerance. This suggests that salt-tolerant microorganisms can respond to and resist high-salt environments by up-regulating the genes encoding their transporters and transport systems, which is supported by similar reports (Zeng et al. 2022). Up-regulation or expression of genes encoding STPs can improve the salt tolerance of cells and prevent the cells from being killed by salt. A salt stress-responsive cysteine protease gene from *Salix matsudana* has been cloned and expressed in *E. coli*, which leads to the enhancement of salt tolerance in *E. coli* (Zheng et al. 2018)*.* By comparing the serine protease genes of the halotolerant cyanobacterium *Halothece* sp. PCC7418 and the freshwater cyanobacterium *Synechococcus elongatus* PCC7942, it has been shown that several serine protease genes of the halotolerant cyanobacterium *Halothece* sp. PCC7418 were drastically up-regulated under salt with high concentration but remained unchanged under salt with low concentration (Patipong et al. 2021). These results suggest that the genes encoding STPs may be associated with salt tolerance in microorganisms.

The adaptation strategy of salt on cell surfaces has also been suggested as one of the reasons for microbial salt tolerance (Mokashe et al. 2018). It has been shown that the fraction of anionic phospholipids in the cell membrane of halophilic bacteria gradually increases with higher salinity levels, resulting in an additional membrane surface charge that helps to maintain the hydration state of the plasma membrane (Mokashe et al. 2018). Compared with the changes in cell membrane composition of yeast tolerant to low and high salt concentrations, it has been found that changes in the composition of saturated and unsaturated fatty acids in highly salt-tolerant yeast lead to a decrease in membrane permeability and mobility, which may contribute to salt tolerance (Qi et al. 2014). Glycerol is commonly used as a microbial protection agent. Under high salt stress, engineered yeast can produce abundant glycerol and secrete it extracellularly. The production of microbial extracellular polysaccharides (EPS) can modify the physical and chemical properties of the membrane, including surface charge and hydrophobicity, facilitating the active transport and diffusion of microorganisms and ultimately enhancing salt tolerance. Current stimulation can promote the production of low molecular weight EPS, which is beneficial for the salt tolerance of microorganisms (Feng et al. 2022). Strain JZ-GX1 can improve its salt tolerance by secreting large amounts of EPS, which was secreted at 6983.9 mg/L at a high salt concentration of 60% (Li et al. 2021a). Furthermore, the addition of exogenous factors such as betaine (Hu et al. 2020a), acyl homoserine lactone (Li et al. 2019), and signal molecule autoinducer-2 (Gu et al. 2021) has been demonstrated to stimulate EPS production and biofilm formation, promoting salt tolerance in cells.

The salt-in strategy is one of the salt-tolerant strategies of microorganisms. This is a process in which inorganic salts (such as sodium and potassium ions) accumulate in the cytoplasmic fluid and involves the entry and exit of metal cations/H^+^ into the cell through membrane proteins (Mokashe et al. 2018). Salt-tolerant microorganisms can respond to salt stress by up-regulating the Na^+^/H^+^ antiporter subunit mnhA-G (Zeng et al. 2022). The Na^+^/H^+^ antiporter plays an important role in maintaining cellular pH and cation homeostasis. Microorganisms can expel excess Na^+^ from cells driven by ATP (Edbeib et al. 2016). Several anti-transporter genes have been reported (Fujisawa et al. 2005). A CPD photolytic gene, PnPHR1, has been isolated from the Antarctic moss, *Pohlia nutans* (Wang et al. 2021b). The gene enhances the salt tolerance of the microbe by increasing the expression of a high-affinity potassium transporter gene and a tonoplast-bound Na^+^/H^+^ antiporter. In addition, salt-tolerant microorganisms can temporarily increase their cytoplasmic K^+^ content and accumulate osmotic fluid as a more persistent stress response to prevent water loss and survive osmotic and ionic stress (Zheng et al. 2021). Most K^+^ transporters are unidirectional, and osmotic protection of K^+^ entry into cells can only be achieved by balancing the negative charge of other small molecules (Lefebvre and Moletta 2006). Several systems for maintaining K^+^ homeostasis in cells have been reported. These systems, which maintain cell stability, play different roles in different media and transport processes of K^+^ concentration (Lefebvre and Moletta 2006).

The accumulation of compatible solutes is one of the most common salt tolerance mechanisms of microorganisms. In response to a high salt environment, microorganisms accumulate intracellular small molecule substances through intracellular synthesis or direct acquisition from the surrounding media to maintain cellular osmotic equilibrium. These substances are highly water-soluble molecules, known as 'compatible solutes', including glycerol, sugars, polyols, betaine, ectoine, hydroxyectoine, proline, lysine, and glycine (Zhang et al. 2022d). Different microorganisms may use one or more different compatible solutes. The salt stress of marine myxobacteria, *Plesiocystis pacifica* SIR-1 and *Enhygromyxa salina* SWB007, shows that the former only depends on the accumulation of amino acids, while the latter uses hydroxyectoine as the main compatible solute in addition to betaine (Moghaddam et al. 2016; Zhang et al. 2022d). The genetic code also confirms that only the latter has the biosynthesis capacity of betaine, ectoine, and hydroxyectoine. In addition, microorganisms using the intracellular small molecule compatible solute accumulation strategy are generally more flexible and can more easily adapt to pressure or salinity-induced mutation than those that use a salt solution strategy (Kurt-Kizildogan et al. 2017). There may be different mechanisms of salt tolerance between halophilic and halotolerant microorganisms. Researchers have discovered that strict halophilic bacteria employ the salt-in and accumulation of compatible solutes strategies for osmoregulation under high salt conditions. In contrast, halotolerant bacteria utilize the compatible solute accumulation strategy rather than the salt-in strategy to resist salt stress (Vaidya et al. 2018).

## Study on microbial-derived STPs

### Isolation and purification of STPs

Separation and purification of STPs is a complex process. After obtaining suitable enzyme-producing conditions and selecting suitable separation and purification methods, microbial cells need to be cultured to obtain large quantities of the enzyme. Some information on the purification and separation of microbial-derived STPs and their enzymatic properties is collected in Table 2. The steps involved in the isolation and purification of proteases are slightly different due to the different positions of proteases in the cell. For intracellular proteases, microbial cells need to be physically or chemically broken down to release intracellular proteases. Using the above method, a large number of crude enzymes have been obtained (Hou et al. 2019). For extracellular proteases, culture media are usually collected directly and crude enzyme solutions are obtained by concentration and centrifugation (Tanasupawat et al. 2011). Salting-out, dialysis, ion exchange chromatography, and gel filtration chromatography are often used to enrich and purify proteases. The neutral protease with high salt tolerance from *A. oryzae* CICIM F0899 has been purified by ammonium sulfate precipitation, ion exchange chromatography, and gel filtration chromatography (Wang et al. 2013). The total recovery of the enzyme is 2%, and the optimum pH value and temperature are 7.0–9.0 and 50 °C, respectively. The protease shows high salt tolerance in 18% NaCl, maintaining 72% of its initial activity after 14 days. Similarly, a salt-tolerant alkaline protease has been isolated and purified from the culture medium of *A. oryzae* 3.042 by ammonium sulfate precipitation, dialysis, and gel filtration chromatography (Gao et al. 2019). The optimum pH and temperature of the enzyme are 9.0 and 40 °C, respectively. After incubation with 3.0 M NaCl for 7 days, the relative enzyme activity remained above 20%. The same method has been used for the separation and purification of the proteases produced by microorganisms in certain special habitats, such as the ocean, salt farms, and saline-alkali soils. Halophilic neutral proteases from *H. agarilyticus* GUGFAWS-3 have been isolated and purified using dialysis and gel chromatography (Gaonkar and Furtado 2020). The enzyme has activity in the range of 20–80 °C, pH 3.0–13.0, and 0–5.0 M NaCl. The best activity is at 70 °C, pH 7.0, and 3.0 M NaCl.

**Table 2:** Some information on the purification and characterization of microbial-derived STPs reported in recent 8 years

Type of protease	Method of separation and purification	Molecular weight	Salt tolerance	Optimum pH and temperature	References
Alkaline serine protease	Ammonium sulfate precipitation, anion-exchange and gel permeation chromatography	32 kDa	Up to 20% (w/v) NaCl	pH 10.5, 60 °C	Ibrahim et al. 2015a
Alkaline protease	Ammonium sulphate precipitation, anion-exchange chromatography, and gel filtration	35 kDa	Up to 30% NaCl concentration	pH 8.0, 50 °C	Wu et al. 2015
Halo-alkaline serine protease	Acetone precipitation, ultrafiltration and carboxymethyl (CM) cation exchange chromatography	48–50 kDa	2.5 M NaCl, 50% of residual activity	pH 9.5, 35 °C	Ghafoori et al. 2016
Acid protease	Filtration, CM-Sepharose fast flow column, and ultrafiltration	34.5 kDa	3.5 M NaCl, maintaining 60% activity	pH 5.0, 50 °C	Lario et al. 2015
Alkalithermophilic protease	Ethanol precipitation and anion-exchange chromatography	35 kDa	2.7 M NaCl, maximal activity	pH 9.0, 55–56 °C	Abdel-Hamed et al. 2016
Alkaline serine protease	Ammonium sulfate precipitation, dialysis, and DEAE 52-cellulose column	32.3 kDa	30% NaCl concentrations, 18.1% of residual activities	pH 8.0, 40 °C	Xie et al. 2016
Alkaline protease	Ammonium sulfate precipitation, ion exchange chromatography, and gel filtration chromatography	63 kDa	Up to 4.0 M NaCl	pH 8.5, 55 °C	Mokashe et al. 2017
Alkaline protease	DEAE-Sepharose fast flow column and Sephadex G-100 column	47 kDa	0–3.0 M NaCl	pH 8.0, 50 °C	Gao et al. 2017
Alkaline protease	Ammonium sulfate precipitation, dialysis, and Äkta Avant 25 (CM-Sephadex C-50 column, Sephadex G-100 column)	29 kDa	3.0 M NaCl for 7 d, 20% of residual activities	pH 9.0, 40 °C	Gao et al. 2019
Aspartyl aminopeptidase	Ammonium sulfate precipitation, dialysis, and Äkta Avant 25 (Bio-Gel A column, HiTrap Q HP column, superose 6 column)	57 kDa	Beyond 30% of the activity in 3.0 M NaCl	pH 7.0, 50 °C	Gao et al. 2018
Neutral protease I	Ammonium sulfate precipitation, dialysis, and Äkta Avant 25 (DEAE-sephadex A-50 column), hydroxyapatite chromatography	69 kDa	3.0 M NaCl, the activity decreased by 30%	–	Gao et al. 2018
Halo-alkaline protease	Ammonium sulphate precipitation and molecular sieve chromatography	About 37 kDa	Up to 5.0 M NaCl, stable	pH 10.5, 45 °C	Kalwasinska et al. 2018
Metalloprotease	Gel filtration column (Superdex 200 pg) and a HiTrap Q FF ion exchange column	About 35 kDa	20% NaCl for 480 h, 22% of residual activities	pH 6.0–10.0, 25–40 °C	Zhang et al. 2018
Halo-alkaline protease	Ammonium sulfate precipitation, dialysis, and UNO-Q12 anion exchange column	37 kDa	NaCl concentrations (0–2.0 M)	pH 10.0, 70 °C	Daoud et al. 2018
Alkaline serine protease	Ammonium sulfate precipitation, anion exchange (DEAE-Sephadex G-50) and gel filtration (Sephadex G-50) column Chromatography	33 kDa	20% salt concentration, 48.3% residual activity	pH 10.0, 60 °C	Ibrahim et al. 2019
Alkaline serine protease	Ni–NTA Superflow column and dialysis	34 kDa	2.0 M NaCl, 49% relative activity	pH 8.0, 40 °C	Yang et al. 2020
Serine alkaline protease	Ammonium sulfate precipitation, dialysis, and Sephacryl S-200 HR column	30.3 kDa	0.5–5.0 M NaCl, maximum activity	pH 10.0, 65 °C	Ouelhadj et al. 2020
Metalloproteinase	Sephadex-250, ethanol precipitation, dialysis, and Sephadex G-200 gel permeation column	67 kDa	0–5.0 M NaCl	pH 7.0, 70 °C	Gaonkar and Furtado 2020
Alkaline serine protease	Ammonium sulfate precipitation and phenyl Sepharose 6 fast flow column	68 kDa	4.0 M NaCl (w/v), stable	pH 9.0, 70 °C	Chauhan et al. 2021
Serine protease	Ni-agarose column and his-tagged proteins	42 kDa	0.5–4.0 M NaCl, > 75% of maximal activity	pH 9.0, 45 °C	Hou et al. 2021
Halo-alkaline protease	Ammonium sulfate precipitation and phenyl Sepharose 6 fast flow column	70 kDa	2.0–4.0 M NaCl, maximal activity	pH 7.0, 50 °C	Foophow et al. 2022
Alkaline subtilisin	Ethanol precipitation, and Äkta Avant 25 (HiPrep 26/10 desalting column, S-Sepharose FF column)	27.1 kDa	Up to 5.0 M NaCl, stable and active	pH 9.0–9.5, 55 °C	Falkenberg et al. 2022
Alkaline protease	Ammonium sulfate precipitation, phenyl Sepharose 6 fast flow column, and Sephadex G-75 column	40 kDa	0.25–0.5 M NaCl, optimal catalytic performance	pH 10.5–10.0, 50 °C	Raval et al. 2022
Alkaline protease	Ammonium sulfate precipitation, phenyl Sepharose 6 fast flow column, and Sephadex G-75 column	28 kDa	0.25–0.5 M NaCl, optimal catalytic performance	pH 10.5–10.0, 50 °C	Raval et al. 2022

– represents that no relevant information is involved in the reference

Table 2

Multiple strategies have been reported for the purification of microbial proteases, each with a different role (Banerjee and Ray 2017). High-purity proteases can be obtained using these methods or by combining them appropriately. Two alkaline proteases of *Haloalkaliphilic bacterium* D-15–9 and *Oceanobacillus onchorynchii* Mi-10-5_4_, purified by ammonium sulfate fractionation and hydrophobic interaction chromatography, have a certain salt tolerance and temperature tolerance, and the latter is more thermostable (Raval et al. 2022). The halophilic protease Pph_Pro1, cloned from *Pseudoalteromonas phenolica*, has been purified by an osmotic shock procedure and immobilized metal affinity chromatography (Johnson et al. 2018). The purified enzyme has an activity of 0.44 U/mg and exhibits halophilic, alkalophilic, and thermally stable properties. An extracellular protease HlyA from the extremely halophilic archaeon *Halococcus salifodinae* has been isolated and purified by affinity chromatography and gel filtration chromatography (Hou et al. 2021). Its activity is best at 45 °C, pH 9.0, and 1.5–2 M NaCl. It retains the maximum activity of > 75% at a wide range of NaCl concentrations from 0.5 to 4.0 M. With the development of science and technology, label-assisted protein purification technology has gradually become the preferred method for scientific research and large-scale industrial demand (Gomari et al. 2020). Employing fusion proteins can help to save time and cost, and they can help to achieve high-throughput purification of the protein. In practice, high-purity STPs are important due to their high stability and efficiency, thus improving the effectiveness of their applications. Therefore, the isolation and purification of STPs is one of the most important aspects in the study of STPs, and is of great importance in the study of their biochemical properties and applications.

### Salt-tolerant mechanisms of STPs

**Fig. 3:**
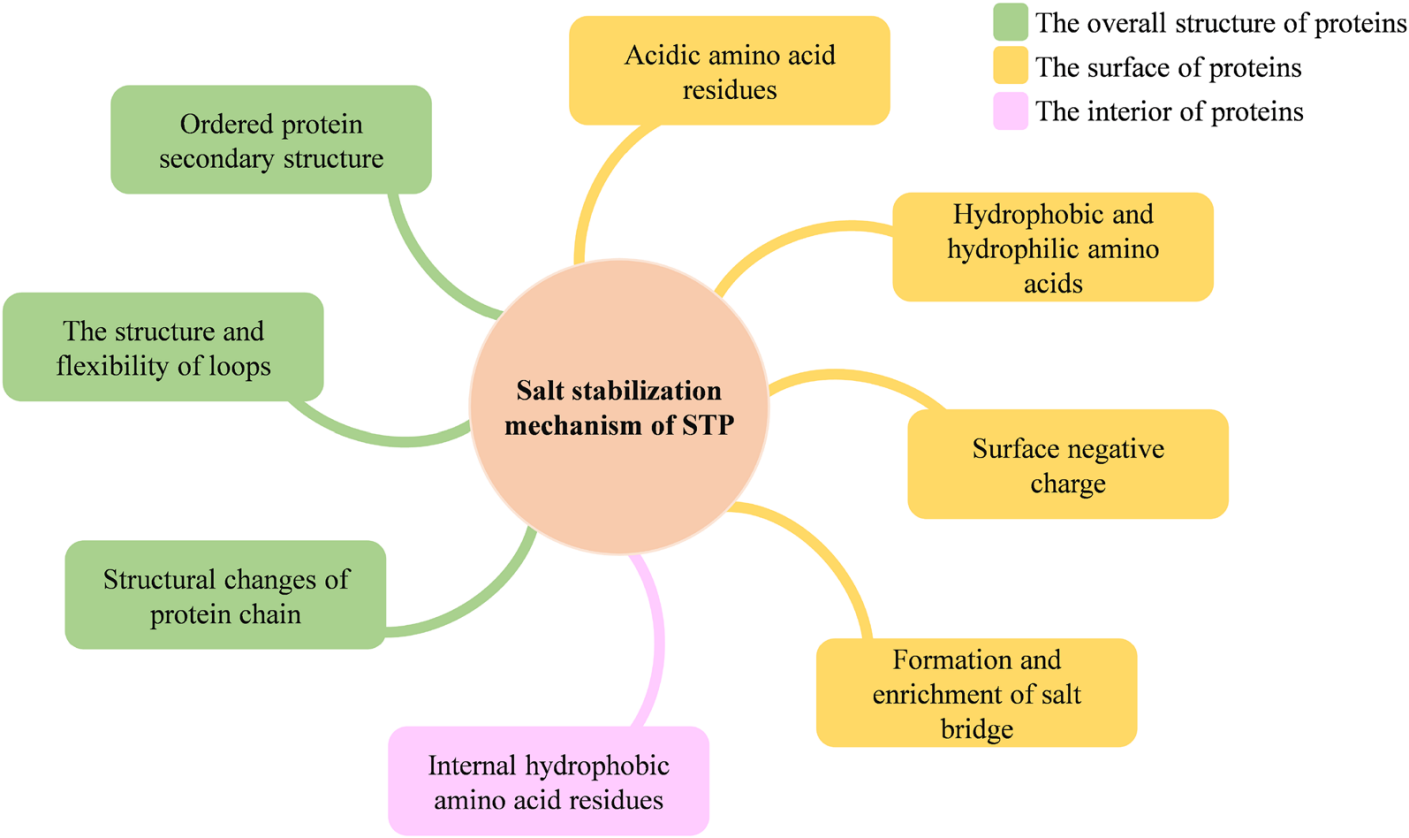
Several mechanisms of salt stability of STPs

High salt concentrations severely affect the solubility, binding, stability, crystallization, and interaction with other substances or proteins, and have a significant impact on the structure and function of proteins (Karan et al. 2012). The salt tolerance mechanism of proteases has been studied more intensively with intensive research conducted in the biological sciences. The salt-tolerant mechanisms of STPs are related to their own secondary structure (low isoelectric point, the flexible loop regions, and ordered structures), surface acidic amino acids, local charge changes, salt bridge formation, and internal hydrophobic amino acid residues (formation of hydration layer). Although halotolerant and halophilic proteases have different salt dependence, this review does not make a strict distinction between halotolerant and halophilic proteases. Various factors of salt stability of STPs are shown in Fig. 3. STPs adapt to high salt environments by following aspects to maintain their catalytic activity and stability.

The ordered secondary structure of proteases (helix, strand, and steering), the structure of the flexible loop region, and the existing form of the protein structure may affect the stability of STPs (Gromiha et al. 1999; Karan et al. 2012). The alpha-helix and beta-strand contents of salt-tolerant acid protease and salt-tolerant neutral protease have been compared (Gao et al. 2018). The results show that the alpha-helix and beta-strand contents of the former are higher than those of the latter, and that the former has a more stable structure. However, a comparison between alkaline proteases and neutral proteases shows that the more stable spatial structure of the former may be due to its higher amount of alpha-helix and beta-strand, but the former has fewer loop regions than the latter (Gao et al. 2019). The ordered structure is not only related to hydrophobicity, but is also influenced by hydrogen bonds and other interactions (Gromiha et al. 1999). Due to their high flexibility, loops in the protein are prone to expansion and instability under adverse external conditions (Ahmad et al. 2012). The instability of loops is mainly due to the interaction between internal residues and the interaction between loop residues and molecules in solution (Vieille and Zeikus 2001). At room temperature, the interaction between Na^+^ and the loop residues is weaker than that of the internal residues (Cai et al. 2022). Some enzymes exist as dimers or tetramers at high salt concentrations, but dissociate into monomers with the decrease of salt concentration, thus losing their activity, such as halophilic malate dehydrogenase (Ebel et al. 1999) and isocitrate dehydrogenase from the halophilic archaea *Haloferax volcanii* (Madern et al. 2004).

The large amount of acidic amino acids on the protein surface helps bind many water molecules and metal ions, increasing the hydration of the protein, thus forming a hydration shell or network. This prevents the protein from aggregating at high salt concentrations, thus maintaining protease activity (Cai et al. 2022; Fukuchi et al. 2003; Takenaka et al. 2022). In contrast to homologous non-halophilic proteins, halophilic enzymes typically contain a relatively high proportion of acidic amino acids, including aspartic acid, glutamic acid, alanine, serine, threonine, and valine (Mokashe et al. 2018). Crystal structure analysis of glucose dehydrogenase from the extremely halophilic archaea *Haloferax mediterranei* has shown a predominantly acidic surface, contributing to the halophilic nature of the enzyme (Britton et al. 2006). This finding is further supported by the analysis of the three-dimensional structure of halophilic malate dehydrogenase and its non-halophilic homologues from the archaea *Haloarcula marismortui* (Dym et al. 1995). Comparison of the amino acid compositions in the whole genomes of halophilic archaea, non-halophilic mesophytic bacteria, and thermophilic bacteria shows that the surface of halophilic proteases contains a significantly higher proportion of acidic amino acids compared to the inner region (Fukuchi et al. 2003). These acidic amino acids account for up to 20–23% of individual proteins and play an important role in the salt stability of proteases (Karan et al. 2012). It has been observed that modifying the amino acid composition on the protein surface can alter the salt dependence of a protein, facilitating cross-over between salt-tolerant and non-salt-tolerant proteins (Tadeo et al. 2009).

Hydrophobic and hydrophilic amino acids on the protein surface also affect the salt-adapted stability of STPs. Hydrophilic proteins have a lower abundance of hydrophobic side chains on their surface compared to non-hydrophilic proteins (Karan et al. 2012). The increase in the number of hydrophilic amino acids compared to hydrophobic amino acids on the protein surface is beneficial for the stability of STPs (Mokashe et al. 2018). A possible explanation for this observation is that the reduction in hydrophobic amino acid content and the corresponding increase in hydrophilic amino acid content facilitates the binding of metal ions to the protease surface charges, forming hydration shells or networks. Additionally, smaller hydrophobic amino acids such as glycine, alanine, and valine show an adaptive mechanism in halophilic enzymes, in contrast to larger hydrophobic counterparts (Mokashe et al. 2018).

The salt adaptation of STPs is influenced by the negative charge on the surface or localized surface of the STPs. For the most part, the effect of salt on protein stability is independent of the total charge of the protein (Karan et al. 2012). The highly negatively charged surface of proteases is a molecular marker of salt adaptation (Hu et al. 2022). The formation of negative charge on the surface of these enzymes is mainly due to the presence of negatively charged amino acids on the surface (Promchai et al. 2018). It has been suggested that the highly negative surface charge of halophilic proteins makes them more soluble and flexible at high salt concentrations, whereas non-halophilic proteins tend to aggregate and harden under these conditions (Ortega et al. 2011). In fact, the negative charge is shielded by more salt, which further prevents the expansion of the protein and maintains the solubility of the protein. Effective hydration and production of ion-pair networks stabilize the structure and activity of enzymes (Mokashe et al. 2018; Promchai et al. 2018). This result has also been confirmed for halophilic alpha-amylase (Hu et al. 2022). These suggest that salt tolerance can be improved by modifying the local charge on the protein surface (Takenaka et al. 2022).

The formation of salt bridges also contributes to the salt tolerance of STPs. The salt bridge refers to the ionic bond between two amino acid residues with opposite charges. In high-salinity environments, the polar groups of protein molecules bind to ions to form salt bridges, which not only play an important role in protein folding, structure and oligomerization, but also enhance the stability of protein molecules (Dym et al. 1995). By comparing the halophilic malate dehydrogenase with its non-halophilic homologue in the archaea *Haloarcula marismortui*, researchers have found that the halophilic enzyme contains more salt bridges and has a higher concentration of surface acidic residues. This difference may explain why the halophilic enzyme is more tolerant to salt (Dym et al. 1995). Molecular dynamics simulations of endoglucanase from *A. niger* have revealed the formation of salt bridges between charged residues and Na^+^ and Cl^−^ ions. These salt bridges affect the activity of the loop and the stability of the pocket, thus giving the enzyme salt tolerance and strong thermal stability in high-salt environments (Cai et al. 2022). Salt bridges can also form between acidic residues and specific alkaline residues on the hydration surface, which enhance the 'rigidity' of the enzyme at high salinity and protect the enzyme from salt ions (Oren and Mana 2002).

Internal hydrophobic amino acids of STPs are also related to their own salt adaptability. Studies have shown that the hydrophobicity of internal amino acid residues of proteins is strongly related to the stability of the internal structure (Gromiha et al. 1999). The greater hydrophobicity of these internal amino acid residues is beneficial for maintaining the internal stability of the protein. One possible explanation for the greater stability of the alkaline protease from *A. oryzae* compared to the neutral protease is that the alkaline protease has a higher molar ratio of hydrophobic amino acid residues (such as alanine, valine, leucine, isoleucine, phenylalanine, and methionine) than the neutral protease (Gao et al. 2019). However, proteins responding to high salt environments often exhibit low levels of hydrophobic and lysine residues (Ortega et al. 2011). Due to the tendency of large hydrophobic residues to form helixes, a significant reduction in the number of these residues may lead to increased flexibility, which may be a factor in salt adaptation (Hu et al. 2022; Paul et al. 2008).

As previously discussed, ordered secondary structures, high surface acidic amino acid content, increased surface negative charge, changes in internal hydrophobicity, and the formation and enrichment of salt bridges seem to be general strategies to improve the salt adaptation of STPs in high salt concentration conditions (Fig. 3). However, these mechanisms may not be universal (Britton et al. 1998), and future studies should further explore the salt-tolerant mechanism of STPs. For some STPs, the presence of salt is essential to restore their active structural conformations (Mevarech et al. 2000). These STPs require the presence of high salt concentrations to exhibit good solubility, stability, and catalytic activity, indicating their dependence on salt (Enache and Kamekura 2010; González-Hernández and Peña 2002; Mokashe et al. 2018). Meanwhile, some STPs do not depend on the presence of salt, but their activity and stability may be inhibited under high-salt conditions (Jothi Basu et al. 2015). Interestingly, due to the employment of multiple salt-adaptation strategies, some STPs not only tolerate or require high salt concentrations. They also maintain their active conformation and good stability in a variety of organic solvents and cataclysmic reagents (Mokashe et al. 2018; Sinha and Khare 2014). The multiple salt adaptation strategy allows STPs to maintain stability and better catalytic activity at high salt concentrations compared to common proteases. Under harsh conditions (such as salt, organic solvents, oxidants, and bleach), the activity of common proteases is inhibited or even denatured, resulting in incomplete hydrolysis of substrate proteins and limited production of small molecule peptides and amino acids (Rodriguez-Rios et al. 2022; Sana et al. 2006). However, under the same conditions, STPs exhibit superior stability and catalytic properties, allowing them to hydrolyze substrate proteins into abundant peptides and small amino acids (Hou et al. 2020; Mokashe et al. 2018). Therefore, due to their outstanding performance under harsh conditions, STPs are a top consideration for applications in the modern biotechnology industry (Liu et al. 2023; Sana et al. 2006).

### Using modern biotechnology to improve the salt tolerance of proteases

While STP-producing microorganisms or new STPs have been discovered from natural environments using various screening techniques, the utilization of proteases should not only focus on the most effective approach to isolate potential enzymes from microorganisms or natural environments, but also consider modifying enzymes through available biotechnology methods to enhance their properties. Various biotechnology techniques have been reported to improve the properties of STPs, such as genetic engineering techniques (Hou et al. 2019), immobilization techniques (Dong et al. 2022), and computer-aided design techniques (Takenaka et al. 2022). These techniques play an important role in improving the activity and stability of enzymes.

Genetic engineering techniques are known to transfer target genes encoding STPs into recipient cells, thereby enabling over-expression of enzymes and enhancing enzyme activity. For example, the serine protease gene *isp* of salt-tolerant *Bacillus* sp. LCB_10_ has been expressed in *E. coli*, leading to highly salt-tolerant proteases (Hou et al. 2019). The enzyme maintains 86% activity in the presence of 7% NaCl. With the development of bioinformatics, genetic engineering techniques are often combined with multi-omics techniques to reveal the salt tolerance mechanisms of STPs and to identify the coding and regulatory genes of microbial STPs. This strategy can increase the chances of discovering new enzymes and enzymes with special functions. These genes are re-fused, recombined, and induced to be expressed in *E. coli*, *Bacillus*, and other microorganisms to improve enzyme production and tolerance. For instance, coding genes from the peptidase S8A subfamily, which has a high ability to hydrolyze proteins, have been screened from the krill metagenomic library using metagenomic techniques (Sun et al. 2020). Subsequently, these genes are expressed in *E. coli*, resulting in the generation of proteases with remarkable salt tolerance. These proteases retain 40% of their residual activity when stored in 3 M NaCl, as demonstrated in previous studies (Purohit and Singh 2013). These STPs have great potential for various industrial applications.

Enzyme immobilization techniques involve immobilizing enzymes on carriers (such as gel, resin, fiber, and metal) to form immobilized catalysts (Ibrahim et al. 2021; Motamedi et al. 2021; Rahman et al. 2016). Adsorption, covalent binding, cross-linking, and entrapment are the four main methods of enzyme immobilization (Dong et al. 2022; Motamedi et al. 2021). These methods not only enhance the catalytic efficiency of the enzyme, facilitating its recovery and reuse, but also enhance its activity and stability (Dong et al. 2022). To improve protease stability, extracellular STPs from *Bacillus* sp. DL-1 have been immobilized by diatomite adsorption and can retain approximately 35.8% of their activity after six repetitions (Dong et al. 2022). In contrast to free proteases, the alkaline protease from *Salipaludibacillus agaradhaerens* immobilized on mesoporous core–shell nanoparticles not only maintains high enzyme activity at high temperatures and low pH, but has been shown to significantly improve enzyme stability against high concentrations of NaCl, organic solvents, surfactants, and commercial detergents (Ibrahim et al. 2021). However, some enzymes may decrease or lose activity after immobilization using these conventional immobilization techniques due to mass transfer limitations and unfavorable conformations between the enzyme and substrate (Altinkaynak et al. 2016; Hanefeld et al. 2013). Using a hybrid nanoflower technique, enzymes can be immobilized by employing proteases and metal ions as organic and inorganic components. This method is highly effective in enhancing the catalytic activity and stability of enzymes, even under harsh conditions such as high salt concentrations, extreme pH levels, and temperature changes (Altinkaynak et al. 2016). This new immobilization technique has been applied to proteases, including trypsin (Lin et al. 2014), papain (Liang et al. 2015), and chymotrypsin (Yin et al. 2015). These cases suggest that these techniques for immobilizing enzymes can be used as potential future approaches for improving the salt tolerance of proteases.

With the development of bioinformatics and the continuous improvement of biological databases (such as protein information resource and protein sequence database), the use of computer-aided rational design of enzyme molecules has gradually gained favor with researchers (Ashraf et al. 2019; Xi et al. 2022; Zhang et al. 2022c). These methods mainly include directed evolution, rational design, and ab initio design (Ashraf et al. 2019). These methods can modify the amino acid sequence (e.g., the surface charge) of proteins by point mutations, insertions, substitutions, and deletions, thus enabling engineered modifications of proteases and enhancing their salt tolerance. Previous studies have reported site-directed mutagenesis of eight site-specific mutations on the surface of moderately salt-tolerant serine proteases from *Bacillus subtilis*, yielding several mutants with substituted aspartic acid and arginine residues (Takenaka et al. 2022). The salt tolerance of the three mutants is 1.2 times higher than that of the wild type. Multiple substitutions of these amino acid residues may alter the ratio of negative to positive charges on the protease surface, thereby increasing the hydration and solubility of the protease surface at high salt concentrations, and improving the salt adaptation of the enzyme itself. Similar studies have also reported that seven amino acid residues of thermophilic proteases are mutagenized into aspartic acid residues by site-directed mutagenesis to introduce a high fraction of negative charges on the protein surface (Takita et al. 2008). The casein hydrolytic activity of six of the seven mutants is 17–19 times higher than that of the wild type when exposed to 4 M NaCl. In addition, directed evolution of random mutagenesis or rational design of site-directed mutagenesis has been employed to improve the thermal stability of proteases (Ashraf et al. 2019; Martinez et al. 2013; Zhao and Feng 2018). These results suggest that computer-aided design techniques can be employed to analyze and predict the amino acid sequence and structural information of proteins, enabling rational modification of protease molecules. These modern biotechnological methods can significantly improve the salt tolerance of STPs, thus providing a more reliable and efficient source of enzymes for industrial applications of STPs.

## Examples of microbial-derived STPs applied to high-salt traditional soy-fermented foods

### Application in soy sauce fermentation

Soy sauce is one of the most popular and important condiments in East Asian cuisine, especially in China and Japan (Liu et al. 2015). In general, there are two main steps in the brewing process of soy sauce, namely, koji making and moromi fermentation (Hu et al. 2020b). The purpose of koji-making is to enrich certain enzyme-producing microorganisms to obtain a variety of enzymes (Zhang et al. 2022b). The quality of koji determines the fermentation efficiency and quality of the soy sauce. The purpose of moromi fermentation is to form a specific flavor of soy sauce and produce fresh flavor ingredients (Wei et al. 2013). *A. oryzae*, which has a strong ability to digest soybean protein, is an important filamentous fungus in the process of making koji in soy sauce (Liang et al. 2009), while *Lactobacillus plantarum* and yeast are used as inoculants in the process of moromi fermentation (Singracha et al. 2017). *A. oryzae* is rich in alkaline and neutral proteases, but less active in acid proteases (Gao et al. 2010). Given that the brewing process of soy sauce is a low pH and high salt system, the alkaline proteases in *A. oryzae* gradually lose their activity under acidic conditions, so neutral proteases dominate in this phase (Hu et al. 2020b). In general, most extracellular proteases of *A. oryzae* are salt-intolerant and are rapidly inactivated during the first few days of moromi fermentation, resulting in a decrease in raw material utilization and flavor content of soy sauce (Gao et al. 2020).

Various strategies have been used to improve the ability of *Aspergillus* to produce STPs in soy sauce production. Through N^+^ ion implantation mutagenesis, a mutated strain A100-8 of *A. oryzae* 3.042 with improved protease production has been derived, exhibiting an approximately 44.1% increase in salt-tolerant acid protease activity (Zhao et al. 2012). *A. oryzae* 3.042 has also been modified using atmospheric pressure and room temperature plasma techniques (Gao et al. 2020). The obtained mutant H8 has high STP activity and is able to significantly increase the amount of peptides and free amino acids in soy sauce. Similarly, the expression of several key hydrolase genes from *Aspergillus sojae* or *A. oryzae*, such as protease, peptidase, and glutaminase, in the salt-tolerant yeast *Zygosaccharomyces rouxii* not only increases the yield of STPs, but also serves as a potential means to provide additional hydrolase during soy sauce fermentation (Yuzuki et al. 2015).

In addition to genetic modification, exogenous addition of STPs has been shown to be effective in improving protein utilization in soy sauce brewing ingredients and soy sauce fermentation quality (Chen et al. 2023). Previous studies have reported the purification of various STPs from *A. oryzae*, including salt-tolerant acid protease (Lee et al. 2010), salt-tolerant neutral protease (Wang et al. 2013), salt-tolerant alkaline protease (Gao et al. 2019), and salt-tolerant aspartate aminopeptidase (Gao et al. 2018). The STP from *A. oryzae* LK-101 is relatively stable at pH 4.5–7.5, below 40 °C, and up to 10% salt concentration, which suggests that it may perform various functions in soy sauce and even fish sauce fermentation, especially to improve flavor by hydrolyzing protein substrates (Lee et al. 2010). The salt-tolerant neutral protease derived from *A. oryzae* CICIM F0899 shows robust salt tolerance up to 18% NaCl and significant thermal stability under saline conditions (Wang et al. 2013). Furthermore, results from a small pilot scale of 0.5 to 5.0 L using the enzyme in soy sauce production have shown promise for the enzyme in soy sauce production. The STP from *A. oryzae* 3.042 exhibits greater stability under high-salt conditions compared to non-STPs, and its presence is strongly associated with soy sauce quality and raw material utilization (Gao et al. 2019). Salt-tolerant aspartyl aminopeptidase is capable of specifically releasing fresh-flavored glutamate and aspartic acid with low threshold values from peptides containing glutamate and aspartic acid residues at the N-terminus of soybean proteins, which plays a key role in fermented soybean products (Gao et al. 2018; Stressler et al. 2016). Remarkably, salt-tolerant aspartyl aminopeptidase from *A. oryzae* 3.042 demonstrates excellent salt tolerance and holds great promise for applications in the production of soy sauce, soy paste, sufu, and tempeh (Gao et al. 2018). Glutamyl transpeptidase is another pivotal enzyme involved in glutamate production during high salt fermentation of soy sauce by *A. oryzae*. The gamma-glutamyl transpeptidase derived from *Aspergillus sydowii* exhibits high activity even under high-salt conditions (Senba et al. 2023). It has also been shown to increase the levels of the fresh flavor amino acid L-glutamate in soy sauce. Immobilized *B. subtilis* bacteria with a novel salt-tolerant L-glutaminase have been applied to high-salt dilute soy sauce brewing, resulting in a 45.9% increase in L-glutaminase levels (Zhang et al. 2022a). Collectively, these salt-tolerant proteases play a key role in soy sauce fermentation by accelerating fermentation, increasing raw material utilization, enhancing product flavor, shortening the production cycle, and reducing production costs.

### Application in soy paste fermentation

Soy paste generally refers to one of the condiments made from soybeans, broad beans, and other bean bases mixed with salt water and spices in a certain container after a long fermentation. Its name varies across regions: Dajiang in Northeast China, Doenjang in South Korea, and miso in Japan (Chun et al. 2020; Yue et al. 2021). Like soy sauce, soy paste undergoes a long fermentation period of 2–6 months. During the fermentation phase, enzymes produced by microbial growth and reproduction are used to break down proteins, starches, and other components in the raw material. These transformations produce peptides, free amino acids, and sugars, resulting in the distinctive flavor and color of soy paste (Zhou et al. 2021).

*Aspergillus* species, especially *A. oryzae*, are the main microorganisms used in soy paste fermentation, producing a variety of STPs (Ao et al. 2018). Currently, many strategies such as the selection of excellent enzyme-producing microorganisms and the co-fermentation of multiple strains have been employed to expedite the fermentation cycle, reduce production costs and enhance the flavor profile of soy paste. By subjecting *A. sojae* to successive mutagenesis using several conventional mutagenic breeding methods, the mutant EUN13 has been obtained, exhibiting a 3.8-fold increase in STP yield compared to the parent strain (Lim et al. 2019). Co-cultivation of *A. oryzae* QM-6 with *A. niger* QH-3 has been shown to enhance STP activity in Pixian broad-bean paste, resulting in high protease activity in broad-bean koji (Tang et al. 2020). Ninety-seven strains have been isolated from broad beans, and 16S rDNA, ITS (internal transcribed spacer), and 26S rDNA sequences have been analyzed based on morphological classification. Bacterial strains have significantly higher protease and peptidase activity compared to yeast and molds (Zhou et al. 2021). However, simulations of the fermentation of broad bean paste by two salt-tolerant fungi and two salt-tolerant bacteria have shown that the total protein, peptide, and amino acid content is higher in the fungal group than in the bacterial group (Lin et al. 2022). Fungal-derived enzyme systems have demonstrated greater albumin and glutenin activity under neutral conditions. These variations may arise from differences in the physical and chemical properties of STPs from different sources, resulting in different hydrolysis conditions and different properties of the final peptide product (Tang 2017).

The synergistic fermentation of soybean residue and soybean meal by adding of exogenous proteases and specific strains has shown promising results (Heng et al. 2022). This method stimulates the growth of fermenting microorganisms and increases the content of organic acids, ultimately improving both fermentation efficiency and substrate utilization. These studies show that synergistic fermentation of soy paste using STPs and microorganisms promotes the availability of a rich system of salt-tolerant enzymes. This beneficial combination leads to improved fermentation efficiency and enhances the overall flavor quality of the product. Similarly, combining STPs with *Lactobacillus brevis* GABA 100 and* A. oryzae* KACC 40250 effectively accelerates the production of aminobutyric acid and flavonoid glycosides in Korean doenjang, significantly reducing the manufacturing time of the product (Li et al. 2017). Salt-tolerant aminopeptidase from marine *B. licheniformis* can effectively improve the hydrolysis and debittering efficiency of soybean isolate protein (Lei et al. 2017). *A. oryzae* capable of producing L-leucine aminopeptidase and STPs are obtained by conventional stochastic mutagenesis, which can be used as industrial fermenters for the production of meju, doenjang, and ganjang, as well as novel fermented soy-based sauce-like products (Lim et al. 2019). Salt-tolerant serine proteases isolated from commercial *A. oryzae* KSK-3 have also been used not only in miso brewing, but also as natural preparations for oral fibrinolytic therapies and nutraceuticals (Shirasaka et al. 2012).

### Application in sufu fermentation

Sufu, a traditional high-salt bean food, is made by mixing tofu with brine and fermenting it with microorganisms. It is rich in proteins, lipids, peptides, amino acids, fatty acids, and flavor compounds such as alcohols, ketones, and aldehydes (Han et al. 2001). The fermentation process of sufu is complex, and its unique flavor and nutritional value are influenced by the type and metabolic activity of the microorganisms involved in the fermentation process (Li et al. 2021b). The microflora determines its safety, smell, and nutritional quality (Li et al. 2021b). Through the analysis of the core functional microbiota related to flavor compounds in the eight stages of naturally fermented bean curd, the results have shown that nine types of bacteria (*Bacillus*, *Enterobacter*, *Lactobacillus*, *Sphingobacterium*, *Stenotrophomonas*, *Tetragenococcus*, *Trabulsiella*, *Unclassified*, and *Weissella*) and six species of fungi (*Alternaria*, *Sterigmatomyces*, *Actinomucor*, *Fusarium*, *Debaryomyces*, and *Candida*) are identified as the core functional microbiota. These microorganisms can significantly affect the production of flavor compounds in the process of natural production process (He and Chung 2020). During fermentation, the metabolism of these microorganisms converts macromolecular proteins and fats into small molecular compounds such as peptides, amino acids, and fatty acids (Li et al. 2021b).

In addition to these specifically functional microorganisms, the fermentation of sufu also relies heavily on salt-tolerant enzymes, primarily proteases, α-amylases, and lipases (He et al. 2022). In fact, the fermentation process of sufu mainly involves biochemical and physical changes involving the degradation of proteins, lipids, and sugars (Chou and Hwan 1994). It has been shown that these chemical changes in the fermentation process of sufu, especially in the ripening process, are affected by sodium chloride and ethanol (Han et al. 2003). The addition of alcohol to the brine solution not only preserves but also slows down the hydrolysis of proteins during aging (Chou and Hwan 1994). These factors contribute to the long production cycle (usually 3–6 months) of sufu like other high-salt soy-fermented foods, which limits its industrial production (Feng et al. 2014; Han et al. 2004).

In recent years, there has been a surge in research efforts to expedite the production cycle of sufu. Fermentation with multiple strains rather than a single strain, such as *Mucor* and *Rhizopus* hybrid fermentation. *Mucor* can secrete abundant STPs and other beneficial enzymes, while *Rhizopus* can produce small amounts of ethanol and organic acids, and its growth temperature is lower than that of *Mucor*. Therefore, *Rhizopus* is used in the fermentation process of sufu, which breaks the seasonal limitation of sufu production and reduces the amount of ethanol in sufu fermentation (Wang et al. 2020). The introduction of a mixed starter consisting of yeast and lactic acid bacteria has been shown to be beneficial in enhancing sufu fermentation (Xie et al. 2018). This approach not only increases the activity of salt-tolerant enzymes such as protease, lipase, peptidase, and α-amylase but also contributes to the enrichment of free amino acids and other functional components, resulting in sufu with distinct characteristics in terms of color, aroma, taste, and functional substances. Taking advantage of its ability to thrive and its abundance of salt-tolerant enzymes in low-temperature environments, *Aspergillus flavus* has been employed in cryogenic fermentation of sufu, effectively addressing the limitation of commercial sufu production at low temperatures (Cheng et al. 2009).

Furthermore, the addition of exogenous salt-tolerant enzymes is widely utilized as a convenient approach in commercial applications. This approach enhances the enzymatic system of fermenting microorganisms and capitalizes on the synergistic effect between microorganisms and enzymes. As a result, it not only streamlines the production process and reduces the production cycle, but also enhances product quality and environmental hygiene. Studies have reported that the addition of STP as an adjuvant to the fermentation process of Kedong sufu (a typical sufu fermented using bacteria) (Feng et al. 2014). The levels of water-soluble proteins, amino acid nitrogen, peptides, total free amino acids, and 11 out of 17 free amino acids are significantly higher compared to the control group without enzyme addition, indicating that the ripening of sufu can be accelerated and the ripening time of sufu can be shortened by adding STPs. Similarly, accelerated ripening by exogenous proteases has also been demonstrated in cheese (Kilcawley et al. 2012). In addition, the use of STPs has been shown to be effective in enhancing the flavor and product quality of Tofu-misozuke (a food inherent to the Fukuoka district of Japan is a type of soybean curd fermented in miso) (Funaki et al. 1997). Mold-fermented sufu and enzymatically ripened sufu (by soaking salted tofu cubes in the prepared koji mash containing various salt-resistant hydrolases) are two common processes used in sufu making. Studies have reported that the fermentation cycle of koji enzyme-ripened sufu typically takes only about two to three weeks compared to that of mold-fermented sufu, which is much shorter than the four to six months required to prepare mold-fermented sufu (Feng et al. 2014; Li et al. 2010). This suggests that the rate of hydrolysis by the addition of salt-resistant enzymes, especially STPs, is higher than that of mold-fermented sufu during the production of sufu.

## Conclusion

Due to their specificity, STPs have gradually been recognized as promising candidates for applications in various fields. The improvement of salt tolerance of proteases based on various modern biotechnologies needs to be established on the basis of elucidating the mechanism of salt tolerance of proteases. However, salt-tolerant mechanisms of STPs from different microbial sources are unclear. Whole genome sequencing, transcriptome sequencing, and proteome sequencing of salt-tolerant microorganisms have been performed using bioinformatics methods to understand their genome structure, biosynthesis, and metabolic pathways, and to explore the types of genes associated with STPs. The relationship among salt-tolerant microorganisms, genes involved in STPs, and STPs should be clarified. Exploring the interplay between protease sequence structure, spatial conformation, functional properties, and salt stress, and elucidating the salt tolerance mechanisms of STPs from different microbial sources is a research focus. Visual analysis and protein engineering techniques can be combined to achieve this.

In addition, low yield, poor stability, and limited catalytic efficiency are also issues that need to be addressed in the context of STPs and their application to high-salt traditional fermented foods. Given the low yield of salt-tolerant enzymes, one important research direction is to use a combination of traditional microbial screening techniques and modern molecular biology techniques to rapidly, accurately, and efficiently screen and identify microorganisms from more ecological environments that can produce abundant STPs. Another way to address the problem of low microbial enzyme production is to use multi-omics techniques to mine the salt-tolerant genes of salt-tolerant microorganisms in combination with high-efficiency heterologous expression systems to rapidly obtain low-cost STP products. Due to the poor stability and limited catalytic efficiency of STPs, appropriate immobilization methods or techniques can be chosen to construct stable and efficient immobilized STPs to improve the rate of reaction between enzyme and substrate matrix, enzyme stability, and multiplexing. In addition, rational design and modification of STP molecules based on modern biotechnology to improve enzyme activity and stability is one of the future research directions for STPs. Of course, various computer-aided techniques (such as protein database and model construction, big data analysis and processing, artificial intelligence prediction and accurate identification) can help further explore the physiological and metabolic properties of STPs and salt-tolerant microorganisms to improve production efficiency and output quality. In the future, STPs will be used more and more widely in various fields. At the same time, methods to improve the properties of STPs will continue to be innovated, which will lay a solid foundation for their greater usefulness in practical applications.

## Data Availability

Not applicable.
